# Transcriptome Analysis of Wheat Roots Reveals a Differential Regulation of Stress Responses Related to Arbuscular Mycorrhizal Fungi and Soil Disturbance

**DOI:** 10.3390/biology8040093

**Published:** 2019-12-11

**Authors:** Catarina Campos, Tânia Nobre, Michael J. Goss, Jorge Faria, Pedro Barrulas, Mário Carvalho

**Affiliations:** 1 ICAAM—Instituto de Ciências Agrárias e Ambientais Mediterrânicas, Instituto de Investigação e Formação Avançada, Universidade de Évora. Pólo da Mitra, Ap. 94, 7006-554 Évora, Portugal; tnobre@uevora.pt (T.N.); jmsf@uevora.pt (J.F.); mjc@uevora.pt (M.C.); 2 School of Environmental Sciences, University of Guelph, Guelph, ON N1G 2W1, Canada; mgoss@uoguelph.ca; 3 Laboratório HERCULES, Universidade de Évora, Largo Marquês de Marialva 8, 7000-809 Évora, Portugal; pbarrulas@uevora.pt

**Keywords:** *Triticum aestivum*, arbuscular mycorrhizal fungi, transcriptomic response, soil disturbance, manganese stress

## Abstract

Symbioses with soil microorganisms are central in shaping the diversity and productivity of land plants and provide protection against a diversity of stresses, including metal toxicity. Arbuscular mycorrhizal fungi (AMF) can form extensive extraradical mycelial networks (ERM), which are very efficient in colonizing a new host. We quantified the responses of transcriptomes of wheat and one AMF partner, *Rhizoglomus irregulare*, to soil disturbance (Undisturbed vs. Disturbed) and to two different preceding mycotrophic species (*Ornithopus compressus* and *Lolium rigidum*). Soil disturbance and preceding plant species engender different AMF communities in wheat roots, resulting in a differential tolerance to soil manganese (Mn) toxicity. Soil disturbance negatively impacted wheat growth under manganese toxicity, probably due to the disruption of the ERM, and activated a large number of stress and starvation-related genes. The *O. compressus* treatment, which induces a greater Mn protection in wheat than *L. rigidum*, activated processes related to cellular division and growth, and very few related to stress. The *L. rigidum* treatment mostly induced genes that were related to oxidative stress, disease protection, and metal ion binding. *R*. *irregulare* cell division and molecular exchange between nucleus and cytoplasm were increased by *O. compressus*. These findings are highly relevant for sustainable agricultural systems, when considering a fit-for-purpose symbiosis.

## 1. Introduction

The symbiosis that is established between the soil-borne arbuscular mycorrhizal fungi (AMF) and roots of most land plants can provide many different agroecosystem services, such as an efficient use of soil nutrients, protection against biotic and abiotic stresses, and improved soil aggregation [1,2]. Recently, the importance of AMF for crop sustainable production has gained recognition and it has been shown that the association between wheat (*Triticum aestivum* L.) and AMF can positively affect plant phenotypic plasticity by increasing nutrient uptake [3] and the plant’s ability to cope with adverse conditions, such as high salinity [4,5], drought [6], or soil metal toxicity [7,8]. Thus, it is clear that the association of wheat with microorganisms affects its phenotype and it adds a source of variability that can have crucial effects on the functionality of the organism considered as a whole—the holobiont [9,10]. The holobiont is defined as the host organism and all of its associated symbiotic microorganisms, being the hologenome the summation of the genetic information of the host and its microbiota [11]. Accordingly, the putative contribution of other associated microorganisms, like bacteria, on the holobiont phenotype is not considered, when referring to wheat and its AMF symbiotic community. However, due to the known importance of AMF and impact in this system, we are arguably dealing with a most significant component of the holobiont. AMF can be horizontally transmitted through extraradical mycelial networks (ERM) and mycelial proliferation in the soil greatly extends the area over which plant roots may acquire soil phosphorus (P) and other major and micronutrients [12]. Individual plants can simultaneously associate with multiple species of AMF and *vice-versa*, but they can still exhibit considerable selectivity in their associations [13,14].

Manganese (Mn) is a micronutrient element essential for normal plant growth and development. Like other micronutrients, roots absorb Mn and distribute it throughout the plant to sink organelles (plastids and mitochondria) and storage sites (vacuole). It is integral for most photosynthetic organisms as a component of the oxygen-evolving complex in photosystem II, serving as an enzyme cofactor in the water-splitting reaction for producing oxygen and providing electrons to the photosynthetic electron transport chain [15,16]. Mn can, in some cases, be replaced by other metals as a cofactor, typically magnesium (Mg) [17,18]. In contrast, when Mn is in excess, it can replace Mg and inhibit enzymatic reactions, which can have detrimental effects on the processes in which Mg is involved [19]. Manganese toxicity has long been recognised as an important factor that can limit plant growth [20]. The accumulation of oxidised Mn in the cell wall leads to the appearance of brown spots on mature leaves, which then result in chlorosis, and root can exhibit brown colour and cracks [21]. Large Mn content in the soil can be further problematic in the context of global climate warming: there are evidences that, in crop plants, including wheat, increasing ambient temperature can enhance leaf Mn uptake [22,23].

Manganese tends to predominantly accumulate in the plant shoots than in the roots, as demonstrated in Mn labeling experiments with ^54^Mn at an early stage of wheat development, where fast Mn transport from roots to shoots was observed in the xylem and it was essentially immobile in the phloem [24]. It was also found that the Mn-induced limitation to wheat growth largely depends on the Mg/Mn ratio and not on the absolute concentration of manganese [25]. Magnesium reduces Mn uptake and it also reduces the proportion of manganese translocated from the roots to shoots [25].

The potential for wheat bioprotection against excessive manganese through the mutualistic symbiosis with AMF that is naturally present in the soil has been previously investigated. An intact extraradical mycelium (ERM) was found to be more effective than other forms of propagule (e.g., spores) in providing protection against stress to a host plant. In a soil causing Mn toxicity, the growth of wheat increased when the species yellow-serradella (*Ornithopus compressus*), rather than ryegrass (*Lolium rigidum*), was grown prior to wheat, provided that the soil was undisturbed and, therefore, an intact ERM was present at wheat seeding [7]. However, Mn concentration in the shoots remained similar, regardless of the previous plant. Hence, the ability of the AMF to exclude Mn from the plant could not explain these results [7]. More recently, it was found that the AMF communities of wheat grown in an undisturbed soil are closely related to that of the preceding host species (*O. compressus* or *L. rigidum*), and different to the one found when soil was previously disturbed or not cropped [26,27]. Furthermore, it was also found that the molecular responses in wheat to microbial colonization were specific to the host–symbiont interaction. Thus, in soil causing Mn toxicity, plant genes from the symbiotic pathway responded to ERM disruption and to the different AMF communities established [27].

There are some studies where plant transcriptional responses to different single AMF species or AMF consortia were compared [28,29,30], but most of the studies have only evaluated plant transcriptional responses by inoculating with one AMF isolate and comparing the plant response without the fungus (see for example reference [31]). In natural conditions, the plants interact with a multitude of AMF species. Therefore, studies focusing on the molecular responses to different AMF communities are needed if we want to predict ‘in field’ dynamics.

In this study, we aimed to understand the response mechanisms of wheat to two different preceding plant species known to induce different AMF communities in wheat roots and provide different degree of protection against soil Mn toxicity [7,27]. Analysis of the transcriptomewas performed in the roots of wheat planted after *Lolium rigidum* or *Ornithopus compressus*, with and without soil mechanical disturbance (where the ERM is completely fragmented) prior to wheat planting (Disturbed vs. Undisturbed), at one and five weeks post-planting, while using the same roots as were used in our previous study [27]. Amongst others, by qPCR, we validated some plant genes involved in Mn transport (NRAMP family) and EIN2, an essential positive regulator of ethylene signaling, which is known to be affected in metal exposed plants [32]. Furthermore, and because there are very few examples where the fungal response has been concomitantly measured with the host [33,34], we studied the transcriptome of *Rhizoglomus irregulare* (formerly *Rhizophagus irregularis* [35]), which was known to be present in the roots of wheat that were subjected to the present transcriptomic analysis (see [27]). We focused on this AMF species, as it is the main model with publicly available genome and transcriptome sequences [36,37,38]. In addition, we showed previously that the preceding plant species did not significantly affect the number of operational taxonomic units (OTUs) of the genus *Rhizophagus* (now *Rhizoglomus*) in the roots of wheat [27]. Therefore, we were not dealing with an *a priori* condition where some treatments were colonized more by this species than others. We were particularly interested in understanding the molecular mechanisms responsible for the higher tolerance to Mn of wheat when preceded by the species *O. compressus*. We hypothesized that there is considerable transcriptional variation of genes in the plant and the fungal partner that is manifested in associations of variable benefits to the plant.

## 2. Material and Methods

### 2.1. Plant Material and Treatments

The plant material was obtained through a two-phase greenhouse experiment designed to analyse wheat (*T. aestivum* L., var. Ardila) transcriptome in response to two different preceding mycotrophic plant species known to provide different degrees of protection against soil Mn stress (*O. compressus* or *L. rigidum*) [39] and two soil disturbance regimes: Disturbed vs. Undisturbed [27]. We used an unsterilized sandy loam Eutric Cambisol soil that was obtained from a natural pasture and known to give rise manganese toxicity to wheat, as described by Brito et al. [7].

In phase 1 of the experiment, *O. compressus* or *L. rigidum* seedlings were planted and grown in 8 L pots over eight weeks. Six pots were used for each preceding plant system, with three plants per pot. The plants were grown under the natural light/dark regime. At the end of phase 1, the plant shoots were excised. The soil was passed through a 4 mm sieve in half of the replicates of each system, thus creating an AMF source comprising spores and fragmented extraradical mycelium (Disturbed regime). In the remaining pots, the soil was kept undisturbed and, consequently, the mycelial network remained intact (Undisturbed regime) (Supplementary Figure S1).

In phase 2 of the experiment, six wheat seedlings were planted in the same pots as before (Supplementary Figure S1). Living plants of *L. rigidum* or *O. compressus* were never present during this second phase of the experiment, and only roots or root fragments remained (for Undisturbed and Disturbed regimes, respectively). Wheat samples (three plants per pot) were taken at one and five weeks post-planting. At five weeks, the differences in growth between the treatments were visually evident, so we stopped the experiment. The roots were cleaned, washed in distilled water, individually weighted (at five weeks), snap-frozen in liquid nitrogen, and stored at −80 °C for RNA extraction. The shoots were individually weighted (at five weeks), washed in distilled water, snap-frozen in liquid nitrogen, and then grounded for RNA extraction and elemental analysis. The differences in wheat shoot and root weights between the different treatments at five weeks were already presented in our previous study [27].

### 2.2. Element Analysis of Wheat Shoots at Five Weeks

The shoot concentrations of Mn, Mg, phosphorus (P), potassium (K), and calcium (Ca) were determined through inductively coupled plasma mass spectrometry (ICP-MS). The dried shoot samples (50 mg, pooled from three plants from each pot) were pre-digested overnight with 2 mL of Suprapur HNO_3_ (67–69%, Fisher Chemicals, Waltham, MA, USA). The samples were then heated to 110–120 °C for 24 h and 0.5 mL of Suprapur H_2_O_2_ (30%, Merck, Darmstadt, Germany) was added to further digest the organic material. The process was repeated until a clear solution with no precipitates was obtained. The digested material was resuspended in a 2% Suprapur HNO_3_ solution (50 mL) and kept at 4 °C until analysis.

Elemental composition was determined through ICP-MS in an Agilent 8800 ICP-MS Triple Quadrupole system (Agilent, Santa Clara, CA, USA). Ruthenium (Ru), rhodium (Rh), and iridium (Ir) were used as the internal standards. Each sample was processed in triplicate. One digestion blank and two certified reference material (NIST SRM 1573a, tomato leaves; and, NCS ZC73030, wheat) were included in each digestion batch, to check the accuracy and estimate the detection limits of the method. Elemental concentration was correlated with shoot weight through exponential correlation while using R program language version 3.5.3 (https://www.r-project.org/).

### 2.3. RNA Isolation and Illumina Sequencing

Total RNA extraction was performed on individual roots while using the Maxwell^®^ 16 LEV simplyRNA purification kit (Promega, Madison, WI, USA), according to the manufacturer’s instructions. RNA was quantified using a NanoDrop-2000C spectrophotometer (Thermo Fisher Scientific, Waltham, MA, USA) and its integrity was checked by gel electrophoresis. From two pots of each disturbance regime, samples from three plants were pooled while using equal amounts of RNA. RNA quality was analysed with BioAnalyzer Agilent DNA High Sensitivity kit. All of the RNA-seq libraries were constructed using the Truseq stranded mRNA sample prep kit (Illumina, San Diego, CA, USA) and paired-end sequenced (2 × 150 bp) on an Illumina HiSeq 4000 system at Genoscreen (https://www.genoscreen.fr/fr/).

### 2.4. Mapping Procedures

The paired end-reads from raw sequence data were trimmed while using Trimmomatic [40] with default settings. The trimmed reads were imported into CLC Genomics Workbench 11.0.1 (Qiagen, USA) and then mapped against the *Triticum aestivum* cDNA RefSeq Annotation v1.1, available at EnsemblPlants (ftp://ftp.ensemblgenomes.org/pub/plants/release-42/fasta/triticum_aestivum) and containing 133 744 sequences. The settings used were length fraction = 0.8, similarity fraction = 0.8, mismatch penalties = 2, and gap penalties = 3, as set by CLC default parameters. The reads could be mapped back to the wheat transcripts in the 16 samples (Supplementary Table S3). For the *R. irregulare* dataset, we ensured that the sequence reads were not from wheat by using only the reads that did not map to the wheat reference transcriptome, and then mapped them against *R. irregulare* reference transcriptome (DAOM 197198 v2.0) [38]. We have only used the samples from five weeks post-planting for the *R. irregulare* mapping.

### 2.5. Identification of Differentially Expressed Genes (DEGs), Annotation and Gene Ontology (GO) Analysis

The *edgeR* package [40] in R was employed to identify differentially expressed genes (DEGs) from raw counts while using TMM (trimmed mean of M values) as the normalization method, CPM filter = 1, a false discovery rate (FDR) < 0.05 and a Log2 fold change of ≥1 and ≤−1. A pairwise comparison test was performed between Disturbed vs. Undisturbed regimes and between the preceding plant species (*O. compressus vs L. rigidum*). The GO enrichment analysis of the DEGs was performed using the Fisher’s exact test within Blast2GO 5.2.5 software [41]. GO slim (Blast2GO tool) was performed to reduce the complexity of GO terms for the functional analysis of DGEs.

The KEGG Orthology-Based Annotation System (KOBAS) [42,43] was used to perform a further functional classification and pathway assignment of the up- and down-regulated DEGs while using the Blast2GO software.

### 2.6. Construction of Co-Expression Networks

The gene co-expression networks related to disturbance or preceding plant species in wheat genes and *R. irregulare* genes were constructed with the *WGCNA* package [44] within the R environment. The default WGCNA ‘step-by-step network construction’ analysis was used to build modules (clusters of genes displaying similar correlated patterns of transcription). The parameters used were soft-power of seven and minimum module size of 30 genes. The adjacency between genes was calculated and a hierarchical clustering tree with the dissimilarity of the topological overlap matrix was constructed. Similar modules were merged by calculating the module eigengenes, clustering them, and assigning a distance threshold. The module eigengene was calculated in each module and then correlated with the treatment (α = 0.05) to identify modules that were significantly associated with disturbance regime or preceding species in wheat (the later irrespectively of being from the Disturbed or Undisturbed regime). In the weighted gene co-expression network, gene connectivity was based on the edge weight (ranging from 0 to 1) determined by the topology overlap measure, which reflects the strength of the communication between the two genes. The most central and connected genes were considered to be hub genes. Co-expression patterns and interactions of hub genes were exported to and visualized by Cytoscape [45].

### 2.7. qRT-PCR Validation

The same RNA samples as above were used to validate specific genes. The first strand cDNA was synthesized with 500 ng of purified total RNA using the GoScript™ Reverse Transcription System (Promega) using random decamer primers, following manufacturer’s instructions.

Six wheat genes were selected based on its differential expression between disturbance regimes or preceding plant species, being some manganese transporters (NRAMP family), which is of great importance for the present study. The selected genes were *patellin* (*PATL*) (TraesCS1A02G268500.2), *sulphite reductase* (*SiR*) (TraesCS1A02G323400.1), *calmodulin-binding transcription activator 3* (*CAMT3*) (TraesCS4D02G304500.3), metal transporter *NRAMP3* (TraesCS7D02G451900.1), metal transporter *NRAMP5* (TraesCS4B02G300600.1), and *ETHYLENE-INSENSITIVE 2-like* gene (*EIN2*) (TraesCS4D02G036000.3). *EIN2* has homology with the NRAMP family and it is involved in the ethylene pathway and is known to respond to stressful conditions. *PATL*, *SiR,* and *calmodulin-binding transcription activator 3* are also involved in response to stresses, and they were highly differentially expressed between some treatments. One gene encoding glyceraldehyde-3-phosphate dehydrogenase (*GAPDH*) (TraesCS6D02G196300.2), one gene encoding actin (ACT)(TraesCS5D02G132200.1), and one gene encoding for a peptidyl-prolyl cis-trans isomerase (TraesCS6B02G093100.2) were selected among the genes showing stable expression profiles in RNA-seq analyses and they were used as internal reference genes in qRT-PCR analyses. The expression of the metal transporters *NRAMP3* and *NRAMP5* was also evaluated in the shoots. For each gene, specific primers were designed to span at least one intron/exon border to avoid the amplification of potential contaminating genomic DNA, and Netprimer analysed its quality (http://www.premierbiosoft.com/) (Supplementary Table S1).

The quantification of gene expression was performed by RT-qPCR while using 5 μL of 10× diluted cDNA with the Maxima SYBR Green/ROX qPCR Master Mix (2×) (Thermo Scientific) on a 7500 Real Time PCR System (Applied Biosystems, Foster City, CA, USA). The evaluation of expression stability for the reference genes was undertaken while using the statistical application *geNorm* [46]. Relative quantification using the geometric normalisation factors that were obtained from *geNorm* was used to evaluate the expression of target genes. Differences in gene expression between the disturbance regimes and preceding plant species were analysed by *t*-test (α = 0.05).

## 3. Results

### 3.1. Growth and Nutrient Accumulation

The weight of shoot and roots of wheat at five weeks was significantly greater in the Undisturbed regime (*p* < 0.001) than in the Disturbed one (see details in [27]). Within the Undisturbed soil, shoot weight of the *O. compressus* treatment was significantly greater than that in the *L. rigidum* treatment (*p* < 0.05) [27]. No significant differences were found within the disturbed regime for either shoot or roots between the *O. compressus* and *L. rigidum* treatments.

There were no significant differences in the concentrations of Mn, Mg, P, Ca, or K in the shoots of wheat between the *O. compressus* and *L. rigidum* treatments when only considering the Undisturbed regime (Supplementary Table S2). However, when considering all of the samples, the shoot weight increased exponentially with shoot P, Ca, and K concentrations, only decreasing in the case of Mn (*p* < 0.05) (Supplementary Figure S2). The concentration of Mg alone was not significantly correlated with shoot weight (*p* > 0.05); however, the ratio of Mg/Mn, which relates to Mn toxicity, was significantly correlated with shoot weight (*p* < 0.01) (Supplementary Figure S2).

### 3.2. cDNA Sequencing and Aligning on Reference Transcriptome

Sixteen high quality cDNA libraries were prepared while using RNA isolated from roots of wheat at one and five weeks post-planting. Illumina sequencing generated 680 406 440 sequence reads, being 652 994 288 reads retained after trimming and removal of low-quality bases. The percentage of bases with Q ≥ 30 was of 95.70%. Raw sequence reads were deposited in the Sequence Repository Archive (SRA) under the accession PRJNA551129.

Reads from the 16 libraries were aligned to the reference transcriptome of *Triticum aestivum*. An average of 72% of the reads could be mapped back to the wheat transcripts in the 16 samples (Supplementary Table S3). The mapped reads at five weeks post-planting ranged from 33,442 to 315,838 reads for the *R. irregulare* dataset (Supplementary Table S4).

### 3.3. Differentially Expressed Genes at 1 Week Post-Planting

We studied the expression of the 133,744 sequences included in the wheat reference transcriptome. At one week post-planting, 51,680 sequences were retained after CPM filtering and subsequently analysed for the existence of DGEs.

Differentially expressed genes were found between the Disturbed and Undisturbed regimes for both *O. compressus* and *L. rigidum* systems (Supplementary Table S5). In wheat following *O. compressus* there were 223 wheat genes that were up-regulated in the Undisturbed samples and 331 genes up-regulated in the Disturbed ones. The top ontologies in the Undisturbed regime were “regulation of cellular process”, “gene expression”, “protein binding”, “integral component of membrane”, “protein-containing complex”, and “nucleus” (Supplementary Figure S3). In the plants from the Disturbed regime, “protein phosphorylation”, “transport”, “cellular response to stimulus”, “protein binding”, “hydrolase activity”, or “nucleus” were the most represented. A different pattern was observed in wheat after *L. rigidum* (Supplementary Figure S4). In the 434 up-regulated genes that were found in the Undisturbed regime, the GO terms included “gene expression” and “cellular macromolecule biosynthetic process”, but also “response to stress”, “response to chemical”, and “oxidoreductase activity” or “transferase activity”, whereas, in the Disturbed samples, enriched terms comprised “oxidation-reduction process”, “small molecule metabolic process”, or “nucleus” (Supplementary Figure S4).

**Figure 1:**
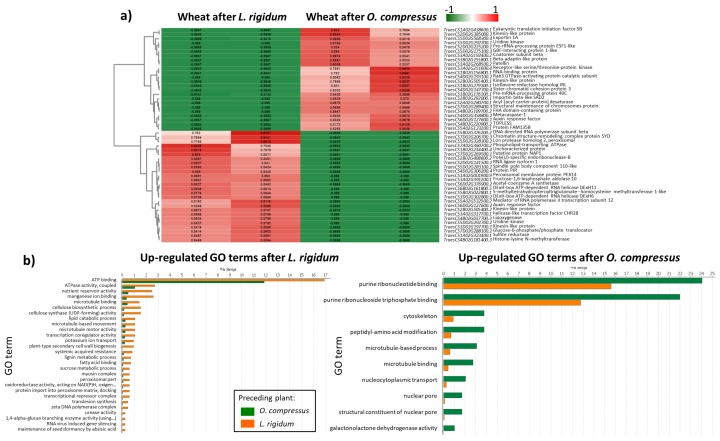
Differentially expressed genes in wheat roots at 1 week post-planting, according to the preceding plant species, in the Undisturbed regime. (**a**) Heatmap of the top 50 differentially expressed wheat genes between the Undisturbed *L. rigidum* and *O. compressus* treatments (green indicates down-regulated genes, red indicates up-regulated genes); and, (**b**) Comparison of the distribution of GO terms in wheat after *L. rigidum* and wheat after *O. compressus*.

The analysis only within the Undisturbed regime, between the *O. compressus* and *L. rigidum* treatments, retrieved 1430 DGEs, with 352 being up-regulated in wheat after *O. compressus* and 1078 up-regulated in wheat after *L. rigidum*. “Purine ribonucleotide binding”, “purine ribonucleoside triphosphate binding”, “cytoskeleton”, “peptidyl-amino acid modification”, and “microtubule-based process” were the most common GO reduced terms in the *O. compressus* treatment (Figure 1). On the other hand, “ATP binding”, “ATPase activity”, “nutrient reservoir activity”, and “manganese ion binding” were the top GO reduced terms in wheat after *L. rigidum* (Figure 1). KEGG pathway mapping revealed that the number of activated pathways were higher in the *L. rigidum* treatment for several metabolic activities, such as “carbohydrate metabolism”, “amino acid metabolism”, or “xenobiotics biodegradation and metabolism” (Table 1; for complete list of activated pathways see Supplementary Table S6).

Among the up-regulated wheat genes in the undisturbed *O. compressus* treatment with high fold changes (logFC > 10) (Supplementary Table S5), we retrieved, for example, genes encoding for putative transporter proteins (importins, potassium transporter, calcium-transporting ATPase, plasma membrane Na+/H+ antiporter), five kinesis-like proteins and two microtubule-associated proteins (play critical roles in mitosis, morphogenesis, and signal transduction), and three nuclear pore complex proteins (facilitate movement of RNA and protein between the nucleus and cytoplasm). In the *L. rigidum* Undisturbed treatment, we found four ATP-binding cassette (ABC) transporters (transport of a wide variety of substrates across membranes in cells), three potassium transporters and one AKT2 potassium channel, and one DExH-box ATP-dependent RNA helicase DExH11 (involved in the regulation of potassium deprivation stress response) (Supplementary Table S5).

### 3.4. Differentially Expressed Genes at Five Weeks Post-Planting

**Figure 2:**
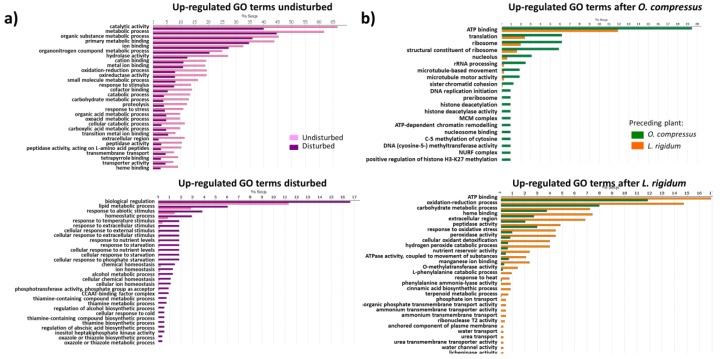
GO enrichment of differentially expressed genes in wheat root at five weeks post-planting, between the Undisturbed and Disturbed regimes (**a**), or according to the preceding plant species (*O. compressus* or *L. rigidum*) in the Undisturbed regime (**b**).

At five weeks post-planting, CPM filtering retained 56,243 transcripts that were firstly pairwise compared between the Undisturbed and Disturbed regimes, irrespective of the preceding plant species. The most represented GO terms in the Undisturbed regime included “catalytic activity”, “metabolic process”, and “organic substance metabolic process” (Figure 2a). KEGG pathway mapping showed that several metabolic activities had a higher number of activated pathways in the Undisturbed treatment than in the Disturbed one (Table 1, Supplementary Table S6), including “carbohydrate metabolism”, “amino acid metabolism”, “lipid metabolism”, “metabolism of cofactors and vitamins”, “biosynthesis of other secondary metabolites”, and “immune system”. The 781 up-regulated genes in the Disturbed regime included GO terms, such as “biological regulation” and “lipid metabolic process”, but also a great number of genes related to “response to external stimulus”, “response to starvation”, or related to overall plant homeostasis (Figure 2a).

Specific genes that were up-regulated in the Undisturbed regime included 16 expansin encoding-genes (Supplementary Table S5), which play a pivotal role in cell wall loosening, thus increasing root cell wall plasticity. Fungal contact triggers the expression of these proteins and it is required for accommodating the fungus in plant root cells [47]. We noted that 44 transcripts encoding for the senescence-specific cysteine protease SAG39, which seems to play a developmental senescence specific cell death function during apoptosis, heavy metal detoxification, and hypersensitive response [48], were also up-regulated in the Undisturbed roots. Additionally, 25 acidic endochitinases, which are involved in root remodelling and growth, were over-expressed in the Undisturbed regime (Supplementary Table S5). The AMF inducible phosphate transporter TRIae;Pht1;10 was also up-regulated in this treatment.

We detected, for instance, 20 transcription factors belonging to the MYB family, which are involved in stress responses in plants [49], 20 disease resistance proteins, eight pathogenesis related-proteins, four protein SAR DEFICIENT 1-like transcripts that are activated in response to biotic and abiotic stresses [50], and the metal-transporter NRAMP3 amongst over-expressed genes in the Disturbed roots. Four metallothionein encoding genes, three heavy metal-associated isoprenylated plant proteins and one metal tolerance protein 1 were also found to be over-expressed in the Disturbed regime (Supplementary Table S5).

When considering the Undisturbed regime alone, we found 418 genes that were up-regulated in wheat roots after *O. compressus* and 1348 that were over-expressed in wheat following *L. rigidum*. 113 GO terms were generated in the *O. compressus* system, and enrichment analysis showed that “ATP binding”, “translation”, “ribosome”, and “structural component of ribosome” were the most common reduced terms, but other up-regulated genes were related to either ribosome or DNA/cell replication (Figure 2b). 122 GO terms were assigned to the 1148 over-expressed genes in the *L. rigidum* treatment. “ATP binding”, “oxidation-reduction process”, “carbohydrate metabolic process”, and “heme binding” were the most common GO reduced terms, but others, such as “response to oxidative stress”, “peroxidase activity”, or “manganese ion binding”, were also up-regulated (Figure 2b). Several metabolic activities that were mapped through KEGG analysis were up-regulated in the *L. rigidum* treatment, including “lipid metabolism”, “metabolism of terpenoids and polyketides”, and “glycan biosynthesis and metabolism” (Table 1, Supplementary Table S6).

We found transcripts involved in resistance to pathogens and protection against stresses (catalase isozyme 2, 26S proteasome non-ATPase regulatory subunit 2 homolog A, protein trichome birefringence-like 28, ATPase family AAA domain-containing protein 5, putative disease resistance protein RGA4) or transcription regulation (mediator of RNA polymerase II transcription subunit 13, transcription initiation factor IIF subunit alpha) among the up-regulated genes with high fold changes (logFC > 10) in the Undisturbed *O. compressus* treatment (Supplementary Table S4). The top expressed genes in the Undisturbed *L. rigidum* system were mostly related to stress responses: fructose-1,6-bisphosphate aldolase 10, microtubule-associated protein, sulfurtransferase, SGT1-1, calmodulin-binding transcription activator 3, heavy metal transporting P1B-ATPase 2, copalyl diphosphate synthase (Supplementary Table S5).

It was notable that only two ABC binding proteins were found to be up-regulated in the *O. compressus* system, whereas 32 were found in the *L. rigidum* one. Furthermore, 30 aquaporin, 30 germin or germin-like transcripts and 30 cytochrome P450 encoding genes were found in the *L. rigidum* system (of these, only one cytochrome P450 was found in *O. compressus*). Forty-two heat shock proteins were also found in the *L. rigidum* system and *L. rigidum* only up-regulated one in *O. compressus*, and 23 glutathione S-transferases. Twenty disease resistance proteins and eight pathogen-related proteins were up-regulated by *L. rigidum*, whereas only six disease resistance proteins were found in the *O. compressus* treatment (Supplementary Table S4). Five phosphate transporters, not described as being inducible by AMF, were up-regulated in the *L. rigidum* treatment. Two auxin response factors and one auxin efflux carrier component were over-expressed in *L. rigidum*, but no auxin-related genes were found in *O. compressus*. Two ETHYLENE-INSENSITIVE 2 (EIN2) genes, which belong to the NRAMP family of metal transporters, were also found to be up-regulated in the *L. rigidum* system, as well as NRAMP5. Seven heavy metal-associated and metal tolerance encoding genes were up-regulated by *L. rigidum*, whereas only one was up-regulated by *O.* compressus. *L. rigidum* also up-regulated the bidirectional sugar transporter SWEET (Supplementary Table S5).

### 3.5. Weighted Gene Co-Expression Network Analysis (WGCNA) at Five Weeks Post-Planting

A scale-free co-expression network using WGCNA was constructed to further explore the impacts of soil disturbance and preceding plant species on wheat transcriptome. WGCNA is a systemic approach that is especially designed for understanding biological networks instead of individual genes. The scale-free topology model fit and the mean connectivity of the network was evaluated over a range of soft threshold powers (β), before selecting β = 8, to confirm that the network was biologically relevant. A dynamic hierarchical tree algorithm was used to divide the clustering tree, resulting in 30 expression modules, ranging from 1351 to 34 genes. The gene co-expression modules showed that module “cyan” was tightly correlated with soil disturbance (Supplementary Figure S5). Hub genes, which have the most connections in a network, were identified in this module and mostly related to “peroxidase activity”, “DNA repair”, “metal ion binding”, and “response to phosphate starvation” (Supplementary Figure S5). GO enrichment analysis of all genes showed that “cyan” was enriched in terms, such as “oxidation-reduction process”, “response to stress”, “cellular response to stimulus”, “oxireductase activity”, or “metal ion binding” (Supplementary Figure S6).

**Figure 3:**
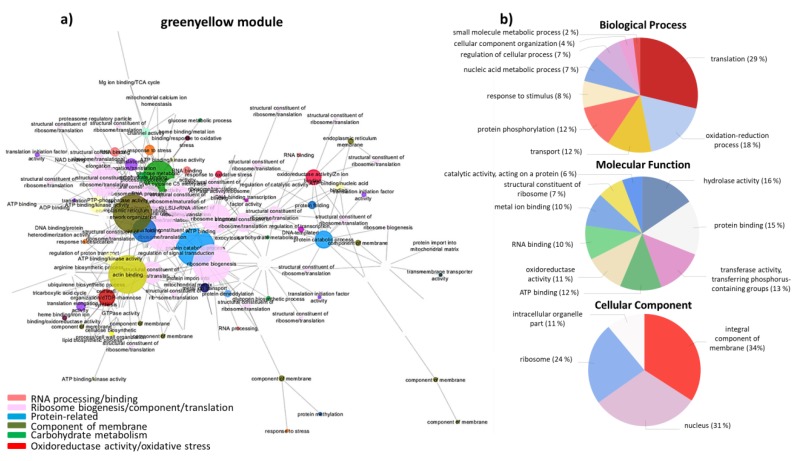
(**a**) Network visualization of interactions between wheat genes in the “greenyellow” module, related to *O. compressus*. Larger nodes correspond to hub genes. (**b**) Pie-charts of GO terms distribution of genes in the “greenyellow” module belonging to the Biological Process, Molecular Function and Cellular Component classes.

**Figure 4:**
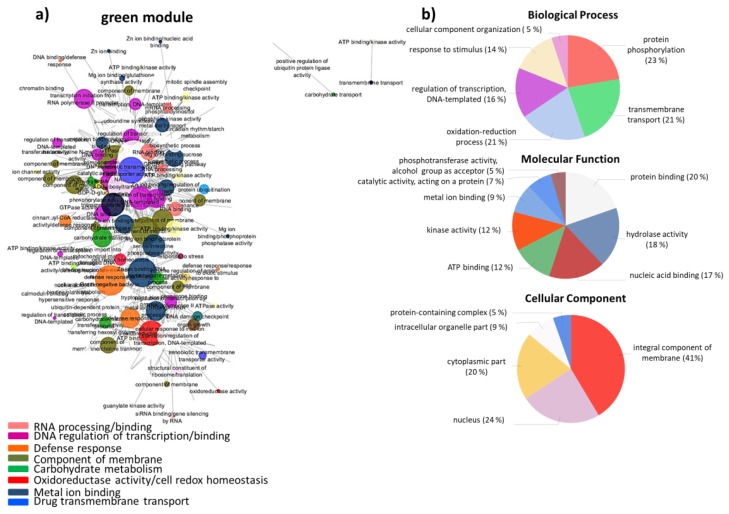
(**a**) Network visualization of interactions between wheat genes in the “green” module, related to *L. rigidum*. Larger nodes correspond to hub genes. (**b**) Pie-charts of GO terms distribution of genes in the “green” module belonging to the Biological Process, Molecular Function, and Cellular Component classes.

The module “greenyellow” was found to be correlated with the preceding species *O. compressus* (Figure 3, Supplementary Figure S7) and “green” was correlated with *L. rigidum* (Figure 4, Supplementary Figure S7). The genes that were present in these modules agreed with the DGEs described above. In the “greenyellow” module, related to the preceding species *O. compressus*, hub genes were related to “ribosome biogenesis/component/translation”, “endoplasmic reticulum tubular network organization”, and “glucose metabolism” (Figure 3). GO analysis of all genes in this module revealed that “translation”, “protein binding”, “RNA binding”, “nucleus”, or “ribosome” were among the most enriched terms (Figure 3).

In the “green” module, which was found to be related to *L. rigidum*, hub genes were related to “protein kinase regulation activity”, “component of membrane”, “drug transmembrane transporter”, “ADP binding”, “regulation of transcription, DNA-templated”, or “oxidoreductase activity” (Figure 4). GO analysis of all genes revealed that “protein phosphorylation”, “regulation of transcription, DNA-templated”, “transmembrane transport”, or “protein binding” were among the enriched terms (Figure 4).

### 3.6. R. irregulare Gene Co-Expression Network Analysis at Five Weeks Post-Planting

The transcriptome of *R. irregulare* in wheat roots was analysed at five weeks post-planting between the Undisturbed *O. compressus* and *L. rigidum* treatments. A scale-free co-expression network using WGCNA was constructed while using a scale-free topology model fit and a soft threshold power β of 7. A dynamic hierarchical tree algorithm was used to divide the clustering tree, which resulted in 36 expression modules ranging from 1402 to 41 genes.

*R. irregulare* hub genes that were related to the preceding plant species *L. rigidum* or *O. compressus* were identified in the “lightyellow” and “orange” modules, respectively (Supplementary Figure S8). GO terms, such as “oxireductase activity”, “6-phosphofructokinase activity” (essential in the glycolysis pathway), “protein binding”, or “ATP binding/kinase activity”, were found as highly interconnected genes in the “lightyellow” network. In the “orange” module we found GO terms, such as “kinase activity/ATP binding/protein phosphorylation”, “protein binding”, and “GTP binding/proteolysis” being highly interrelated with other nodes (Supplementary Figure S8).

**Figure 5:**
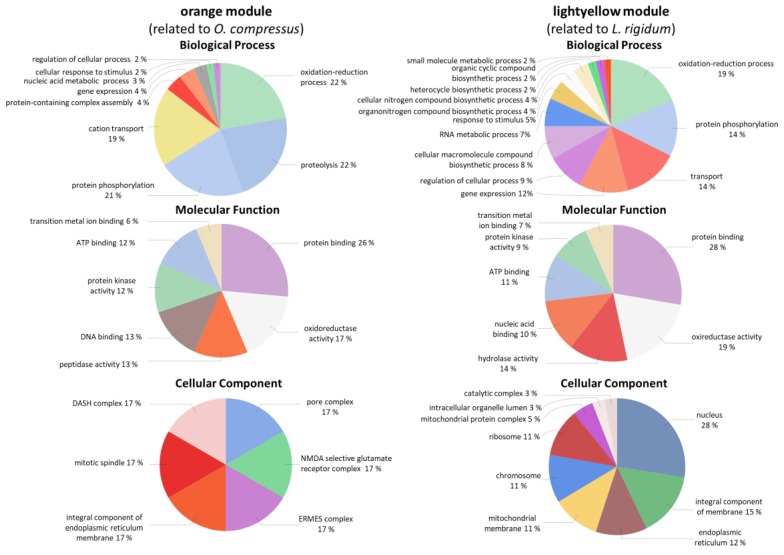
GO enrichment of *R. irregulare* genes in the “orange” and “lightyellow” modules in the Biological Process, Molecular Function and Cellular Component classes. “orange” module shows genes related to *O. compressus*, and “lightyellow” module contains genes connected to *L. rigidum*.

GO enrichment analysis of all genes in the “orange” and “lightyellow” modules showed that they were differentially enriched in terms, such as “cation transport” (19% in “orange” and not found in “lightyellow”), “proteolysis” (22% in “orange”, and not found in “lightyellow”), “heterocycle biosynthetic process” or “cellular nitrogen compound biosynthetic process” (2% and 4% in “lightyellow” and not found in “orange”) (Figure 5). The Cellular Component part was completely different between the two modules: “pore complex”, “mitotic spindle”, “ERMES complex”, and “DASH complex” were found in the “orange” module, whereas terms, such as “nucleus”, “endoplasmic reticulum”, “mitochondrial membrane”, or “chromosome”, were enriched in “lightyellow” (Figure 5). Genes in “orange” module included, for instance, three transcripts encoding a AIG1 domain and also with homology to hedgehog proteins and already described in *Glomus mossae* [51], a Ran binding protein 1, a camk/camkl protein kinase, and four tyrosine kinases specific for activated GTP-bound (Supplementary Table S7). In the “lightyellow” module, it was found a 6-phosphofructokinase encoding gene, two Ypt/Rab GTPase activating proteins, and gene containing BTB/POZ and Kelch domains (Supplementary Table S7).

KEGG enrichment of the “lightyellow” module revealed that four pathways of “carbohydrate metabolism” and four of “amino acid metabolism”, as well as three pathways of the “metabolism of cofactors and vitamins” and two of “energy metabolism” were activated (Supplementary Table S6). In the “orange” module, four metabolic activities were also enriched, but with less pathways involved: “lipid metabolism” (two pathways), “metabolism of cofactors and vitamins” (one pathway), “energy metabolism” (one pathway), and “signal transduction” (one pathway) (Supplementary Table S6).

### 3.7. qPCR Validation

qRT-PCR analysed the expression of six wheat genes selected among those differentially expressed between the Disturbed and Undisturbed samples or between *O. compressus* and *L. rigidum* treatments to validate the root RNA-seq data. As shown in Supplementary Figure S9, all of the genes were differentially expressed as expected, which confirmed the accuracy of transcriptome profiling obtained with RNA-seq.

The metal transporters *NRAMP3* and *NRAMP5*, which were further evaluated in the five week shoots, were found to be differentially expressed both between the Undisturbed and Disturbed regimes in the *O. compressus* system, and between *O. compressus* and *L. rigidum* treatments only within the Undisturbed regime (*NRAMP5*) (Supplementary Figure S9).

## 4. Discussion

Mycorrhizal colonization induces important morphological and functional changes in plant roots, and next generation sequencing techniques can be conveniently used for the analysis of transcriptome profiling during plant/fungi interactions. In recent years, several studies have focused on the effect of mycorrhizal colonization on plant molecular responses (e.g., [31,33,52,53,54]), mostly by inoculating plants with a single AMF species and observing the transcriptomic changes with and without symbiosis. Here, we report the findings from a different approach: with the *a priori* knowledge that different preceding plant species induce different mycorrhizal communities on wheat roots [27], and that different AMF established symbiotic communities provide a differential protection against soil manganese toxicity [7,27], we concomitantly looked into wheat and AMF-derived transcripts to gain a comprehensive view of the regulation of gene expression related to plant tolerance against excess Mn. However, we could only relate that to the transcriptome of *R. irregulare* due to the scarcity of available AMF reference genomes/transcriptomes in the databases. Nevertheless, the present study is, to our knowledge, the first to analyse a scenario that is naturally found in soil—where a mycorrhizal fungus contacts roots of different plants and, *vice versa*, a plant encounters different AMF communities; the combination of these interactions results in different patterns of benefits in the plant–fungal relationship. The implications of our findings are discussed below.

### 4.1. The Effects of Soil Disturbance and Preceding Plant Species Are Observable in 1 Week Plants

Expression changes that could be related to the performance of wheat in a soil conveying Mn toxicity were already visible at one week post-planting. Given that the plant still relies on the energy and nutrients stored in the seed at this stage of development, one might think that soil properties would have little influence on the plant molecular response, but the opposite was observed. In fact, the wheat seedlings transcriptomic response has been found to change, for example, in relation to contrasting nutrient concentrations [55]. GO enrichment analysis showed that “ATP binding”, “ATPase activity”, and “manganese ion binding” were up-regulated in the *L. rigidum* Undisturbed treatment as compared with *O. compressus*. ATP binding cassette (ABC) proteins were found amongst the most up-regulated genes in *L. rigidum* system; ABC transporters family members are known to be engaged in numerous functions, including secondary metabolite transport [56], heavy metal detoxification [57], and phytohormone transport [58], thus being important for tolerance to different kinds of abiotic and biotic stresses. The up-regulation of these transporters in the *L. rigidum* treatment agrees with the Mn stress-induced changes that were later observed in the plants’ growth. Although being an essential element for plants, excess Mn can prevent the uptake and translocation of other elements, such as Ca, Mg, Fe, and P, presumably due to the similarity in ionic radius or binding strength for ligands [59]. This can induce a range of negative effects, including the production of reactive oxygen species (ROS), the exchange of essential metal ions from the active centres of enzymes or binding to functional groups, causing visible symptoms, like chlorosis, necrosis and growth inhibition [60]. In fact, the increased “manganese ion binding” in the *L. rigidum* system indicates an excess of Mn flowing, which could be preventing correct binding of other elements.

### 4.2. Soil Disturbance Increases Phosphate Starvation and Defense Responses in Five Weeks Wheat

Later in wheat development, at five weeks post planting, we found even more striking differences in wheat transcriptomic responses to disturbance regime and to preceding plant species. Soil disturbance created an environment that was clearly negative for plant performance, which was most probably due to the disruption of the previously established network of extra-radical AMF hyphae that extends beyond root depletion zones in search of phosphate [61]. Disrupted mycelia and pieces of colonized roots are not as efficient as intact ERM in colonizing a new host [62]. This fact, being probably connected to other adverse aspects, such as a decrease in the plants’ capability to tolerate biotic and abiotic stresses that can be provided by AMF (see for example [63,64]), led to a sharp decrease in growth and an increase in stress-related responses. In fact, we repeatedly found an enrichment of terms that were related to “response to external stimulus”, “response to phosphate starvation”, “oxidation-reduction process”, “response to stress”, or “metal ion binding” in the Disturbed regime, both in the DGEs and the WGCNA analysis. Phosphate supply was clearly depressed in these plants, which the small concentration found in the shoots further confirmed, compromising plant growth. Generally, the number of activated pathways of many metabolic activities was also reduced in this regime, except for the “xenobiotics biodegradation and metabolism”, “metabolism of terpenoids and polyketides”, and “metabolism of other amino acids”. The disturbance of the rhizosphere seemed to increase the plant need to detoxify or produce secondary metabolites.

One gene family highly expressed in the Disturbed wheat roots was the transcription factors MYB. They are involved in different processes, such as differentiation, hormonal, and biotic and abiotic stresses responses [49,65]. Recently, it was also found that the combined effect of salt stress and AMF mycorrhization led to the up-regulation of these genes in the species *Sesbania cannabina* [66]. Furthermore, the up-regulation of disease resistance and pathogenesis-related genes can indicate that the soil disturbance created a stressful environment where the plant needs to activate defense responses, which are costly to the plant and that could probably be mitigated by an intact ERM [64]. In the Disturbed regime, the disruption of the mycelial network turns spores and colonized root fragments or mycelial fragments into the main propagules’ sources and these must re-enter the entire process of symbiosis establishment with the host. As the initial stages of root colonization by AMF are accompanied by transient induction of plant defenses, followed by localised suppression at later stages of the interaction [67]; this might also play a role in the roots of the Disturbed regime.

“Metal ion binding” was found to be up-regulated in the roots under the Disturbed treatment regime. Plants adopt mechanisms to combat their deleterious effects, such as sequestration in the apoplast or vacuoles, or chelation and formation of stable inactive complexes when manganese or other metals are in excess (see reviews by [68,69]). We found an over-expression of several genes that were related to metal stress, such as the metal chelator metallothionein and the metallochaperones heavy metal-associated isoprenylated plant proteins, indicating a disturbance-related imbalance in metal uptake by the roots. It was also found that the content of Mn in the shoots from the Disturbed regime was larger than from the Undisturbed one, probably meaning that translocation from root to shoot was enhanced. Mycorrhizal fungi can immobilize metals in their biomass, mainly in the cell wall, vesicles, and the glomalin [70]; however, in the Disturbed regime, the ERM network was disrupted and, consequently, the colonization of the roots was less efficient. Therefore, it is likely that these plants were needed to respond to metal stress without much of the benefits that can be induced by AMF. The NRAMP3 Mn transporter, which in rice is located in the plasma membrane [71], was also up-regulated in the Disturbed regime. In rice that was exposed to high Mn concentration, the expression of *NRAMP3* transcript was unaffected, but the NRAMP3 protein was rapidly degraded, which indicated that this transporter is post-translational regulated in response to Mn increase [71]. In the present study, the protein level was not determined, but it is notable that this gene was up-regulated in the *O. compressus* disturbed regime in the aerial part of wheat in comparison with the Undisturbed, but no differences were observed in the *L. rigidum* system. The changes induced by treatments on NRAMP5 were similar. In rice, NRAMP5 has been reported as a major transporter for root uptake of Mn [72], but also for its translocation and distribution in the plant [73]. As the concentrations of Mn in the shoots showed an identical pattern as these genes, they are perhaps not subjected to post-translational degradation in wheat. However, the subcellular localization of these transporters should be investigated in the future to better ascertain their role in Mn flow in wheat. It is also possible that differences in the AMF communities do result in different responses from the plant side and, hence, in different benefits or levels of benefits.

### 4.3. The Preceding Plant Species (O. compressus or L. rigidum) Induces Transcriptomic Changes in Wheat and Arbuscular Mycorrhizal Fungal Partner

The dual approach on AMF colonized wheat roots allowed for us to identify plant and fungal transcripts that were co-expressed at five weeks post-planting. The WGCNA approach clusters gene transcripts and allows for the identification of co-expression patterns to different traits. In this study, we focused on the gene networks existing between wheat or fungi and preceding plant species only in the Undisturbed environment, which is the condition where the wheat bioprotection that is induced by *O. compressus* is evident when comparing to the induced by *L. rigidum*.

The *O. compressus* system activated several processes that were related to cellular division and growth that were evident in the DGEs and in the WGCNA analysis. The amount of up-regulated terms, such as “translation”, “ribosome”, or “RNA binding”, indicates that the plant was allocating its energy for growth, and it did not spend much on other processes, such as defense from diseases or production of secondary metabolites. This was in accordance with the KEGG analysis, as, for example, the number of activated pathways in the “metabolism of terpenoids and polyketides” was reduced when comparing to the *L. rigidum* treatment. The majority of terpenoids, including isoprene, monoterpenoids, sesquiterpenoids, and diterpenoids, are implicated in plant protection against abiotic stress and biotic interactions [74]. In addition, terpenoids also serve as antioxidants that play different roles in plants.

It was evident that the *L. rigidum* treatment failed to reduce the stress in wheat as much as did *O. compressus*. A notable finding was that *L. rigidum* activates transport mechanisms that respond to stress. The up-regulation of ABC binding proteins or aquaporin genes is an example. Aquaporins are membrane channel proteins that were originally discovered as water channels, but their roles in the transport of small neutral solutes, gasses, and metal ions are now well established [75]. One of the aquaporin subfamilies, the tonoplast intrinsic proteins (TIPs), which were also up-regulated by *L.* rigidum, are known to transport glycerol, urea, and ammonia and they are essential for growth under environmental stress [75]. Other aquaporin subfamily, the plasma membrane intrinsic proteins (PIPs) were down-regulated in *Glycine max* and *Lactuca sativa* following AMF infection under drought stress, and mycorrhizal plants accelerated the down-regulation of these genes when compared to non-AMF plants [76]. It has further been demonstrated that the symbiosis with AMF influences the expression of a wide number of aquaporin genes in the host plant subjected to water stress [77]. In our study, no water stress was applied to the plants; however, it is known that mycorrhizal fungi contribute to approximately 20% of the water uptake to the host [78]. It is possible that different mycorrhizal communities have different abilities to uptake water to their host, and that has influenced aquaporin expression.

It is worth noting that, on comparing the Undisturbed and Disturbed regimes, we found an up-regulation of an AMF inducible phosphate transporter in the former; however, five phosphate transporters not described as being directly induced by AMF were up-regulated by *L. rigidum* when comparing *L. rigidum* and *O. compressus* treatments within the Undisturbed regime, but the concentration of P in the shoots of *L. rigidum* and *O. compressus* treatments remained similar. In nature, there is a variation in phosphorus uptake in relation to colonization by different AMF, since the isolates differ in P transfer efficiency and supply to the plant [79,80,81,82]. We can conjecture that the different AMF communities colonizing the wheat roots [27] were not equally efficient in providing P to the plant, and the plant had to increase its P supply by activating phosphate transporters in the *L. rigidum* treatment; it is possible that, to reach those same levels, the plant had to up-regulate its own transporters at the cost of energy for other systems.

The *L. rigidum* system showed increased expression of genes belonging to the NRAMP family: EIN2 and NRAMP5. EIN2 contains an amino-terminal domain with clear homology to the NRAMP proteins. However, its role in transporting Mn is uncertain [83]. What is clear is that EIN2 encodes a protein that is involved in ethylene signal transduction. Biotic and abiotic stresses may modify the pathways for ethylene synthesis or signalling [84,85]. EIN2 is the only gene of all the components involved in the ethylene pathway whose loss-of-function mutations lead to complete ethylene insensitivity [84], and might act in the cross-talk of multiple hormone signalling pathways and responses to pathogens. Interestingly, ethylene insensitive *ein2-1* mutants are more sensitive to metals, which was attributed to an increased metal uptake and a diminished glutathione content, revealing crosstalk between ethylene and the biosynthesis of this antioxidant and metal chelating compound (see review by [32]). NRAMP5, which is involved in Mn transport and was over-expressed in the *L. rigidum* undisturbed system, did not increase the Mn concentration in the wheat shoots of this treatment. *L. rigidum* up-regulated other metal-associated genes when comparing with *O. compressus* and WGCNA showed several hub genes that were related to metal ion binding; this indicates that wheat following *L. rigidum* was under metal stress. The fact that Mn concentration was not greater in wheat after *L. rigidum*, together with the lack of increased the expression of Mn transporters in the vacuole by *O. compressus* treatment suggests that (1) wheat following *O. compressus* was not under metal stress, and it did not need to activate stress responses or metal sequestration or chelation, (2) wheat following *L. rigidum* was under metal stress, had to activate metal-stress responses, such as the glutathione S-transferases, which quench toxic exogenous compounds with the addition of glutathione and protect the cell from oxidative burst [86], probably mitigating Mn stress, and (3) these differential metal stress situations are likely connected to the mycorrhizal communities that colonized wheat roots and that were shaped by the preceding plant species [27].

In this study, we also analysed the transcriptome of *R. irregulare* at five weeks post-planting by mapping the reads against *R. irregulare* reference transcriptome, a species that we previously found that did not show differences in the number of OTUs in wheat roots following *L rigidum* or *O. compressus* [27]. However, we found differences in the repertoire of genes that were related to *O. compressus* or *L. rigidum*. *R. irregulare* has coenocytic hyphae, where a high number of nuclei migrate in a common cytoplasm [87] and can be in contact with multiple plant species at the same time. This situation raises the question as to whether AMF can adjust their transcriptome to the plant species that it colonizes. It was recently shown that the secretome of *R. irregulare* adjusts to host, with some proteins showing differential expression according to the host species [88,89]. With our experimental design it was possible to observe that the effect of the preceding host species was visible, even in a succeeding host species (wheat).

We found, for example, that *O. compressus* enriched “DASH complex”, “pore complex”, and “mitotic spindle” terms. Therefore, it seems that *R. irregulare* cell division and molecular exchange between nucleus and cytoplasm was increased by *O. compressus*. Additionally, “proteolysis” and “protein phosphorylation” were up-regulated by *O. compressus*, mainly by the action of proteins with homology to AIG1 family/hedgehog and protein tyrosine kinases. The expansion of the tyrosine kinase-encoding gene family involved in signalling pathways is a feature of *R. irregulare* [36,37,88]. The AIG1 family, which appears to be involved in plant resistance to bacteria [90], was represented by genes also with homology to hedgehog, and was described as possibly having a role in the development of appressoria during AM symbiosis and as a sensor for plant signals [51].

Glucose is the major carbon form transferred to the AMF at the plant–fungal interface [91]. It is interesting that the gene 6-phosphofructokinase was a hub gene related to *L. rigidum*; this enzyme catalyses the phosphorylation of fructose-6-phosphate to fructose-1,6- bisphosphate, which is a key regulatory step in the glycolytic pathway. The SWEET family of transporters was shown to have differential expression in response to AMF, which suggested that these transporters might regulate sugar export towards the symbiotic interface [92]. We also found the up-regulation of one bidirectional sugar transporter SWEET gene in the *L. rigidum* treatment, which seems to indicate a higher sugar requirement from AMF during symbiosis. In contrast to ectomycorrhizae, where increased flux rates through fungal glycolysis and the tricarboxylic acid cycle were shown in established *Pisolithus microcarpus*/*Eucalyptus globulus* ectomycorrhizas [93], there has been little investigation of carbohydrate metabolism in endomycorrhizal fungi. It is possible that *R. irregulare* from the *L. rigidum* system required more carbon from the plant, and that contributed to the lower growth of the wheat.

## 5. Conclusions

In this study, we identified the molecular responses in wheat and in an arbuscular mycorrhizal fungus partner that could be attributed to the preceding plant species and to soil disturbance regime, factors that are known to affect AMF communities in wheat roots [27]. To our knowledge, this is the first study where such analysis has been done, and it provided clear insights into how the symbiosis can vary in its benefits to the plant. We have shown that these communities result in different transcriptional profiles and symbiont-host interactions that lead to differential fitness of the interaction given a certain stress (in our case, Mn). It was, by far, evident that the *L. rigidum* treatment failed to reduce the stress in wheat, as much as *O. compressus* did, and it was possible to identify the mechanisms that were changed in the plant response. Changes in the *R. irregulare* transcriptome attributable to a preceding host species, which could affect wheat response, were also detected. This findings call for further research, as they can have profound and practical implications for sustainable agricultural systems. It suggests that a fit-for-purpose symbiosis might indeed be promoted once we better understand the system.

## Figures and Tables

**Table 1:** Comparison of activated KEGG pathways between the different wheat treatments at 1 and 5 weeks post-planting.

Number of Activated KEGG Pathways in Each Category						
Metabolic Activities	1 week		5 weeks			
	Undisturbed		Undisturbed vs. Disturbed		Undisturbed	
	*O. compressus* vs. *L. rigidum*				*O. compressus* vs. *L. rigidum*	
Biosynthesis of antibiotics	1	1	1	1	1	1
Carbohydrate metabolism	7	14	14	10	10	10
Nucleotide metabolism	1	2	2	2	2	1
Amino acid metabolism	8	10	10	5	11	10
Metabolism of other amino acids	3	4	3	4	3	4
Lipid metabolism	4	4	9	6	2	10
Metabolism of cofactors and vitamins	3	5	8	3	4	5
Biosynthesis of other secondary metabolites	2	3	7	3	4	6
Xenobiotics biodegradation and metabolism	2	7	4	5	4	4
Energy metabolism	3	5	6	4	2	4
Metabolism of terpenoids and polyketides	0	1	1	2	1	5
Glycan biosynthesis and metabolism	6	5	5	4	1	4
Chemical structure transformation maps	0	0	0	0	0	1
Signal transduction	0	1	1	1	1	1
Translation	1	1	1	1	1	1
Immune system	2	2	2	0	2	2

For list of various metabolic pathways that are grouped under various metabolic activities, please refer to Supplementary Table S6.

## Supplementary Materials

The following are available online at https://www.mdpi.com/2079-7737/8/4/93/s1. Figure S1: Experimental scheme. In Phase 1 of the experiment *O. compressus* or *L. rigidum* seedlings were planted and grown in pots. At the end of Phase 1, the plant shoots were excised. In half of the replicates of each system the soil was sieved (Disturbed regime). In the remaining pots, soil was kept undisturbed. In Phase 2, wheat seedlings were planted in the same pots as before. Living plants of *L. rigidum* or *O. compressus* were never present during this phase of the experiment, only roots or root fragments remained (for Undisturbed and Disturbed regimes, respectively). Figure S2: Elemental concentration was correlated with wheat shoot weight through exponential correlation at 5 weeks post-planting. Shoot weight increased exponentially with shoot P, Ca and K concentrations, decreasing in the case of Mn. The concentration of Mg was not significantly correlated with shoot weight; however, the ratio of Mg/Mn was significantly correlated with shoot weight. Figure S3: Gene Ontology enrichment of the Biological Process, Molecular Function and Cellular Component classes in wheat roots at 1 week post-planting, in the *Ornithopus compressus* system, between the Undisturbed and Disturbed regimes. Figure S4: Gene Ontology enrichment of the Biological Process, Molecular Function and Cellular Component classes in wheat roots at 1 week post-planting, in the *Lolium rigidum* system, between the Undisturbed and Disturbed regimes. Figure S5: Wheat genes at 5 weeks post-planting differentially transcribed between the Undisturbed and Disturbed regimes were clustered into 30 modules using WGCNA. Different colours represent different modules. Dendogram of module-trait correlation showed that module “cyan” was tightly correlated with disturbance. Figure S6: Gene co-expression modules showed that module “cyan” was tightly correlated with soil disturbance. a) Hub genes, which have the most connections in a network, were identified in this module, and were mostly related to “peroxidase activity”, “DNA repair”, “metal ion binding” and “response to phosphate starvation. b) Gene Ontology enrichment of the Biological Process, Molecular Function and Cellular Component classes in the “cyan” module. Figure S7: Scatterplot of Gene Significance (GS) for *Ornithopus compressus* (a) and *Lolium rigidum* (b) treatments vs. Module Membership (MM) in the “greenyellow” and “green” modules. There is a highly significant correlation between GS and MM for both modules. Figure S8: *Rhizoglomus irregulare* gene co-expression network analysis at 5 weeks post-planting. Hub genes were identified in “lightyellow” (a) and “orange” (b) modules, related to *Lolium rigidum* and *Ornithopus compressus*, respectively. Figure S9: The expression of six wheat genes selected among those differentially expressed between Disturbed and Undisturbed regimes or between *O. compressus* and *L. rigidum* treatments were analysed by qRT-PCR. Table S1: Primers used for validation of wheat (Triticum aestivum) selected genes by RT-qPCR. Table S2: Elemental analysis of Mg, Mn, P, K and Ca on wheat shoots at 5 weeks post-planting. Table S3: Read numbers obtained from the Triticum aestivum transcriptome during the bioinformatic analysis. Table S4: Read numbers obtained from the *Rhizoglomus irregulare* transcriptome during the bioinformatic analysis. Table S5: Differentially expressed genes (DGEs) at 1 and 5 weeks post-planting between the different wheat treatments. Table S6: List of activated metabolic pathways in the different wheat treatments and in *Rhizoglomus irregulare*. Table S6: *Rhizoglomus irregulare* genes in the "lightyellow" and "orange" modules.
