# Supplementary material for: Transcriptome Analysis of Wheat Roots Reveals a Differential Regulation of Stress Responses Related to Arbuscular Mycorrhizal Fungi and Soil Disturbance
Source: Biology (Basel). 2019 Dec 11;8(4):93. doi: 10.3390/biology8040093 (PMC6956056; doi:10.3390/biology8040093)
Supplement: Supplementary file 1 [file biology-08-00093-s001.zip › Supplementary Information.pdf]

Supplementary Information

Transcriptome analysis of wheat roots reveals a differential regulation of stress responses related to arbuscular mycorrhizal fungi and soil disturbance

Catarina Campos, Tânia Nobre, Michael J. Goss, Jorge Faria, Pedro Barrulas, Mário Carvalho

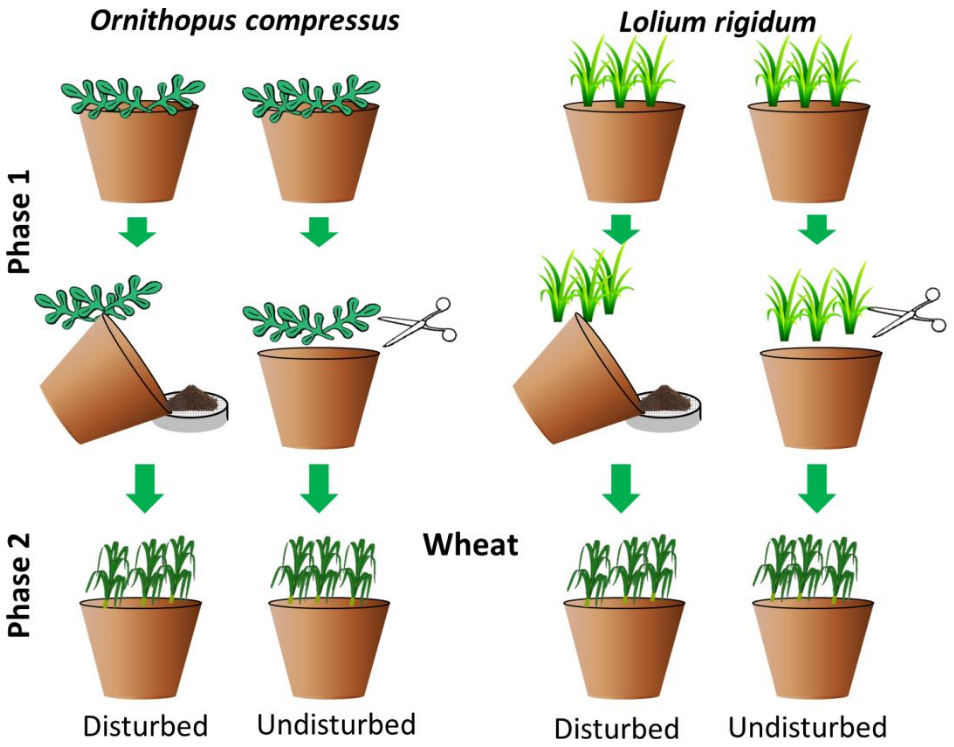

Supplementary Fig. S1. Experimental scheme. In Phase 1 of the experiment *O. compressus* or *L. rigidum* seedlings were planted and grown in pots. At the end of Phase 1, the plant shoots were excised. In half of the replicates of each system the soil was sieved (Disturbed regime). In the remaining pots, soil was kept undisturbed. In Phase 2, wheat seedlings were planted in the same pots as before. Living plants of *L. rigidum* or *O. compressus* were never present during this phase of the experiment, only roots or root fragments remained (for Undisturbed and Disturbed regimes, respectively).

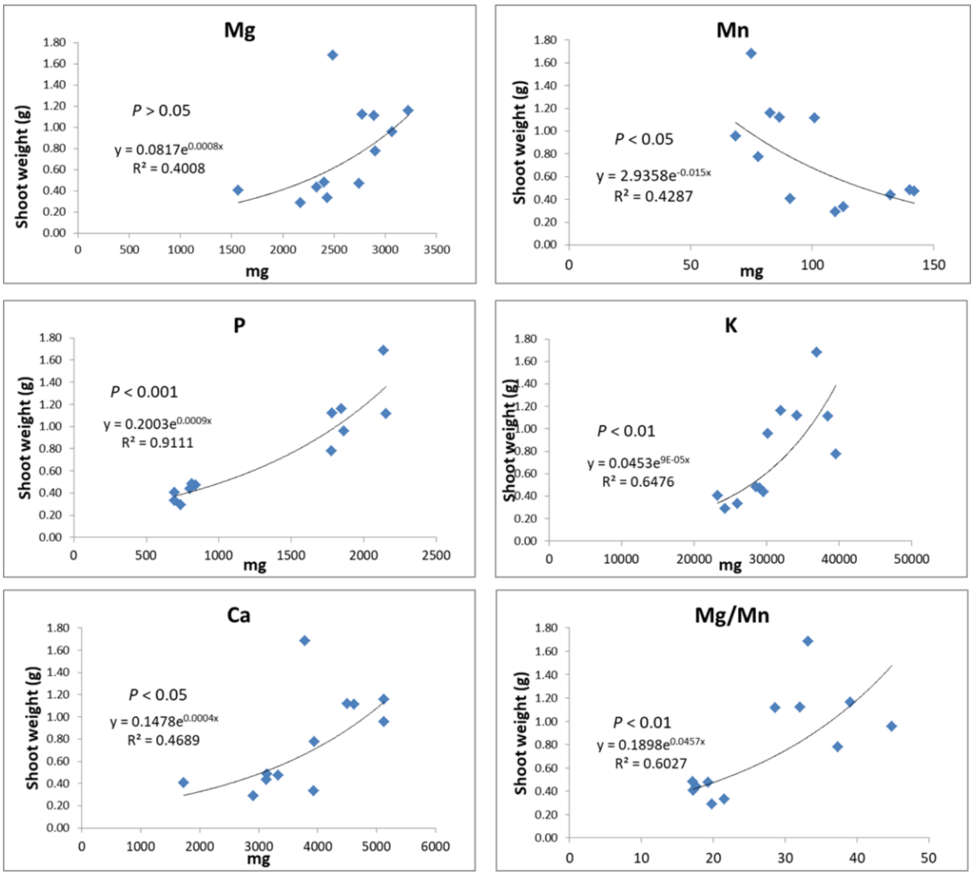

Supplementary Fig. S2. Elemental concentration was correlated with wheat shoot weight through exponential correlation at 5 weeks post-planting. Shoot weight increased exponentially with shoot P, Ca and K concentrations, decreasing in the case of Mn. The concentration of Mg was not significantly correlated with shoot weight; however, the ratio of Mg/Mn was significantly correlated with shoot weight.

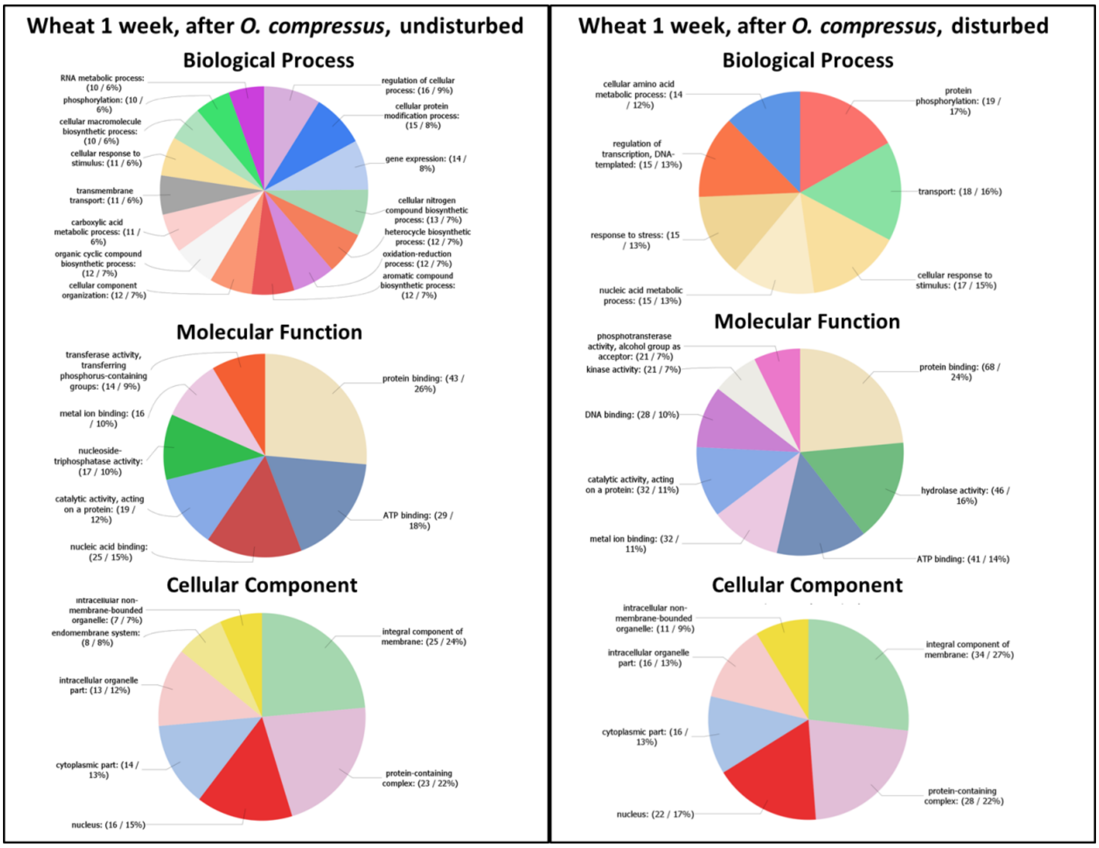

Supplementary Fig. S3. Gene Ontology enrichment of the Biological Process, Molecular Function and Cellular Component classes in wheat roots at 1 week post-planting, in the *Ornithopus compressus* system, between the Undisturbed and Disturbed regimes.

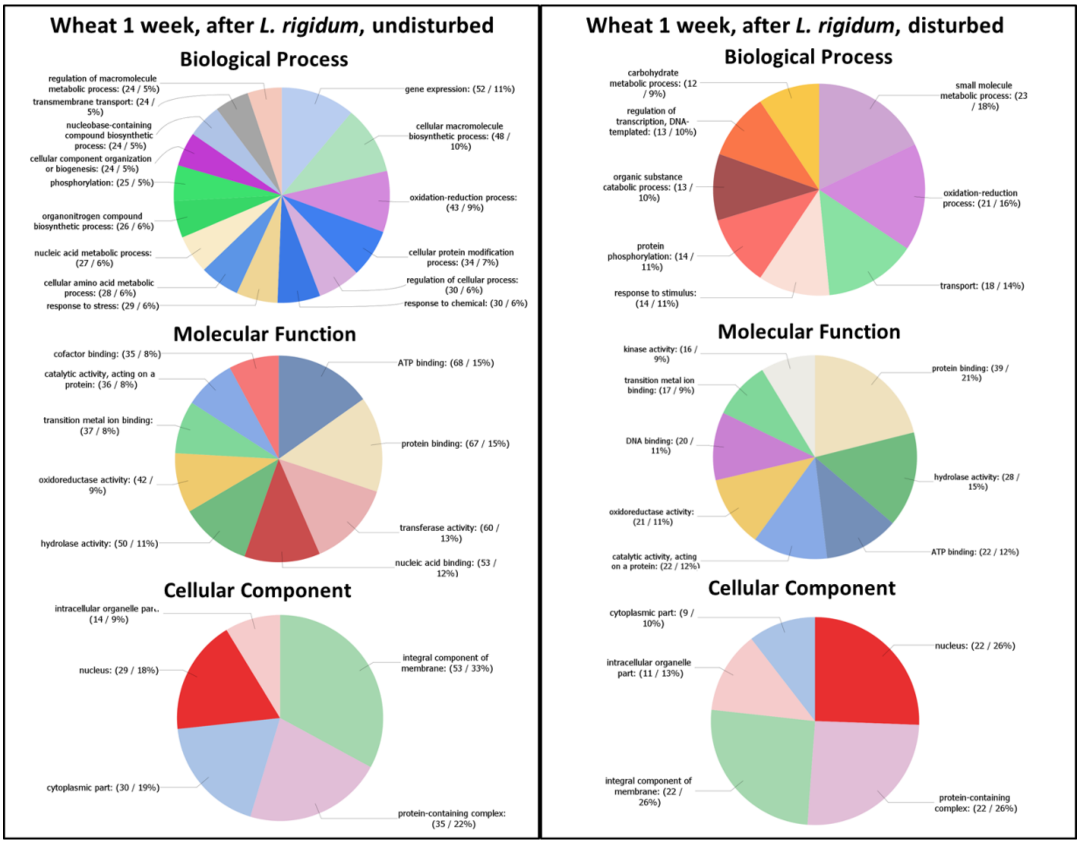

Supplementary Fig. S4. Gene Ontology enrichment of the Biological Process, Molecular Function and Cellular Component classes in wheat roots at 1 week post-planting, in the *Lolium rigidum* system, between the Undisturbed and Disturbed regimes.

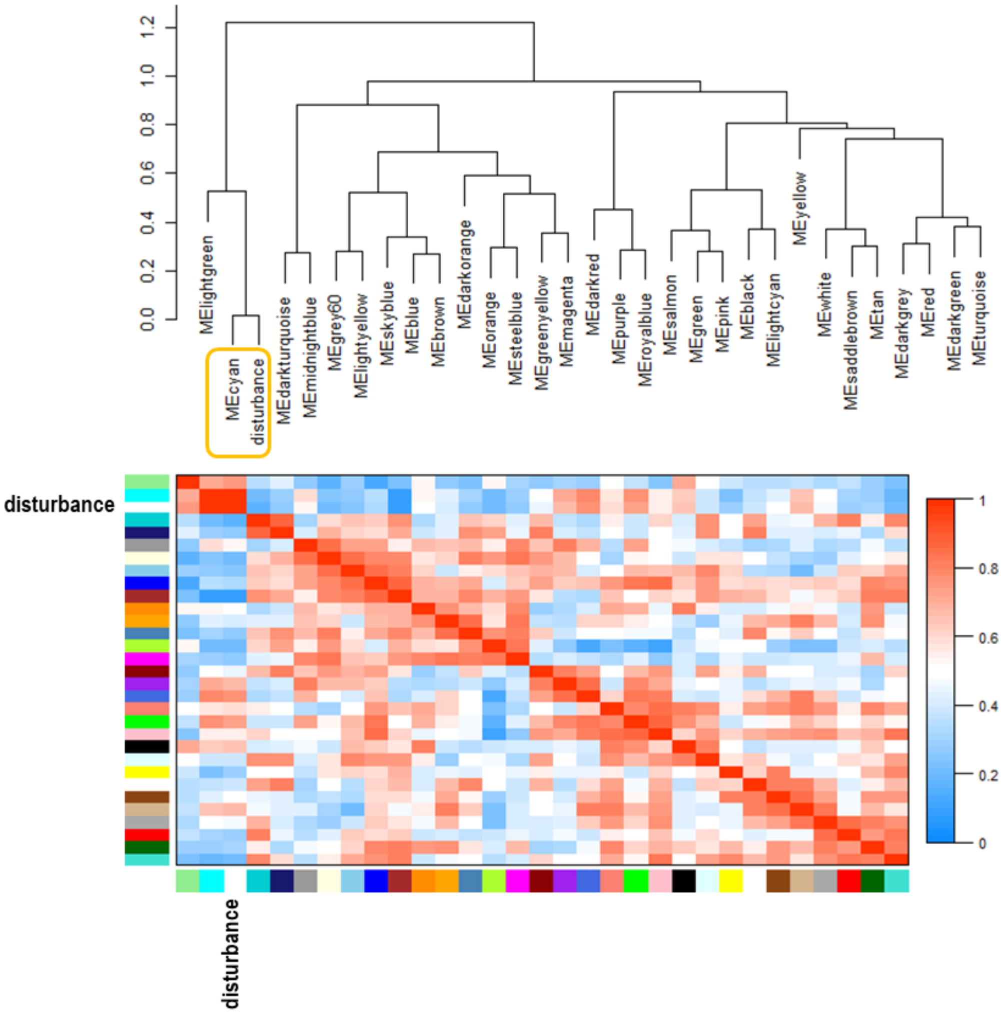

Supplementary Fig. S5. Wheat genes at 5 weeks post-planting differentially transcribed between the Undisturbed and Disturbed regimes were clustered into 30 modules using WGCNA. Different colours represent different modules. Dendogram of module-trait correlation showed that module “cyan” was tightly correlated with disturbance.

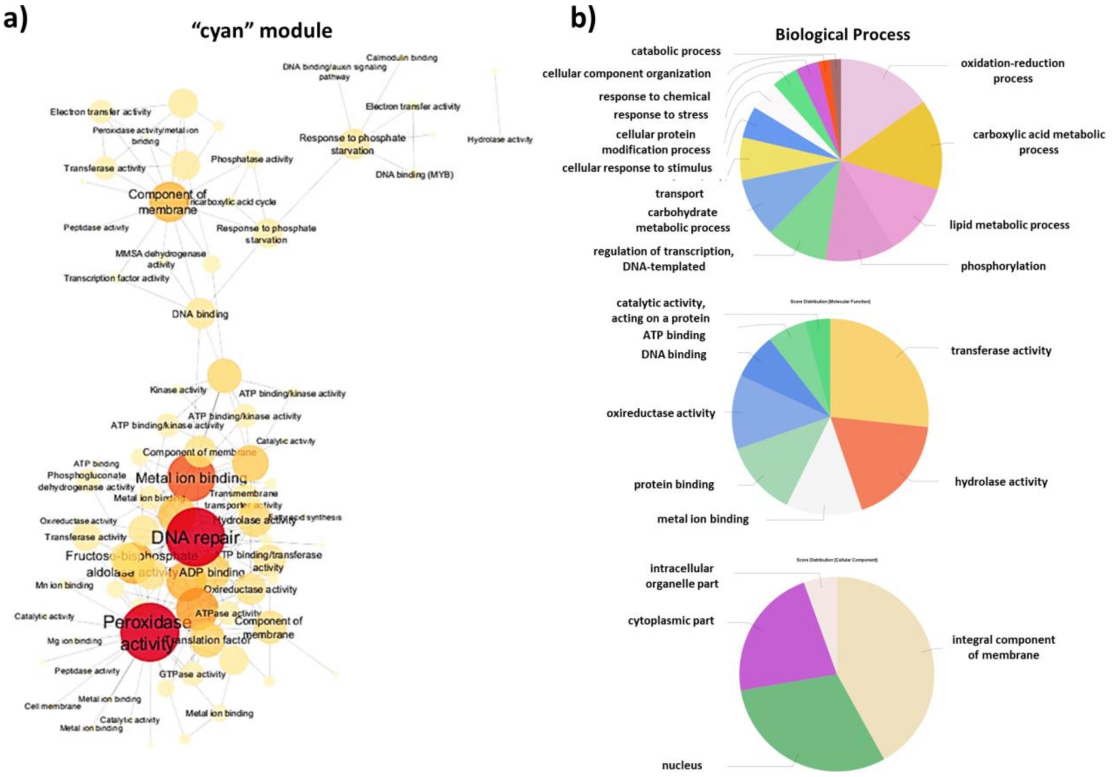

Supplementary Fig. S6. Gene co-expression modules showed that module "cyan" was tightly correlated with soil disturbance. a) Hub genes, which have the most connections in a network, were identified in this module, and were mostly related to "peroxidase activity", "DNA repair", "metal ion binding" and "response to phosphate starvation. b) Gene Ontology enrichment of the Biological Process, Molecular Function and Cellular Component classes in the "cyan" module.

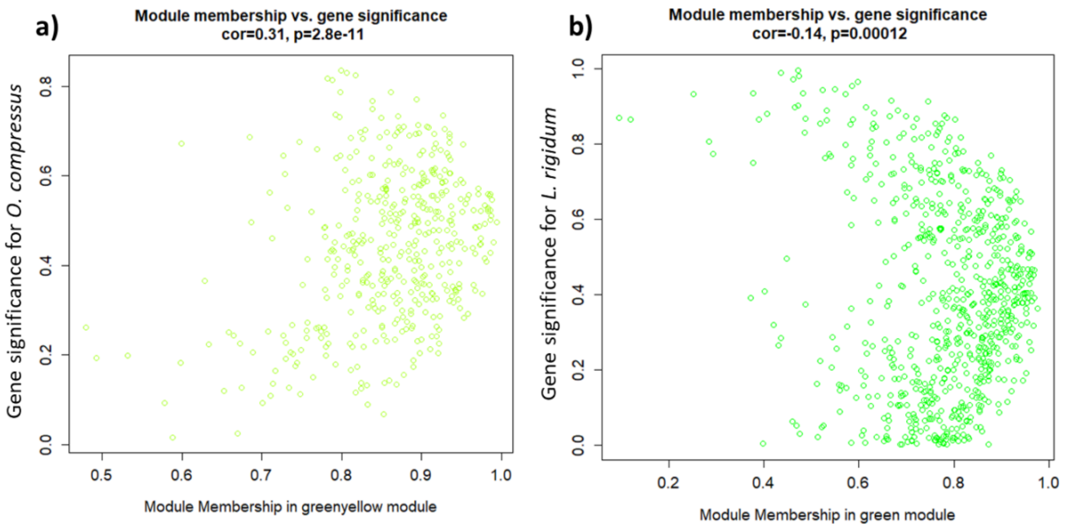

Supplementary Fig. S7. Scatterplot of Gene Significance (GS) for *Ornithopus compressus* (a) and *Lolium rigidum* (b) treatments vs. Module Membership (MM) in the “greenyellow” and “green” modules. There is a highly significant correlation between GS and MM for both modules.

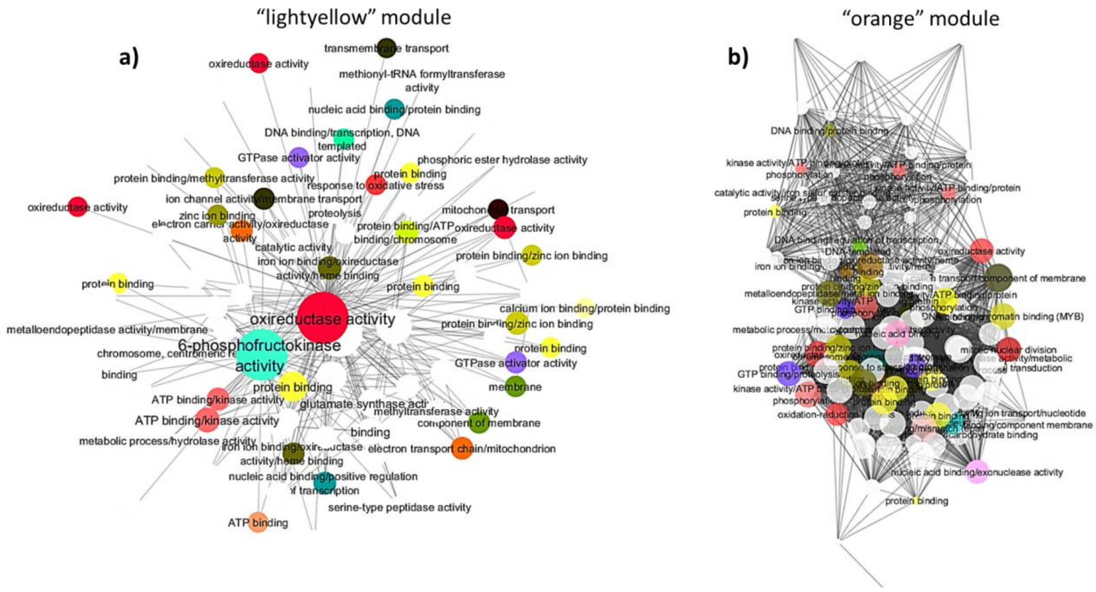

Supplementary Fig. S8. *Rhizoglomus irregulare* gene co-expression network analysis at 5 weeks post-planting. Hub genes were identified in "lightyellow" (a) and "orange" (b) modules, related to *Lolium rigidum* and *Ornithopus compressus*, respectively.

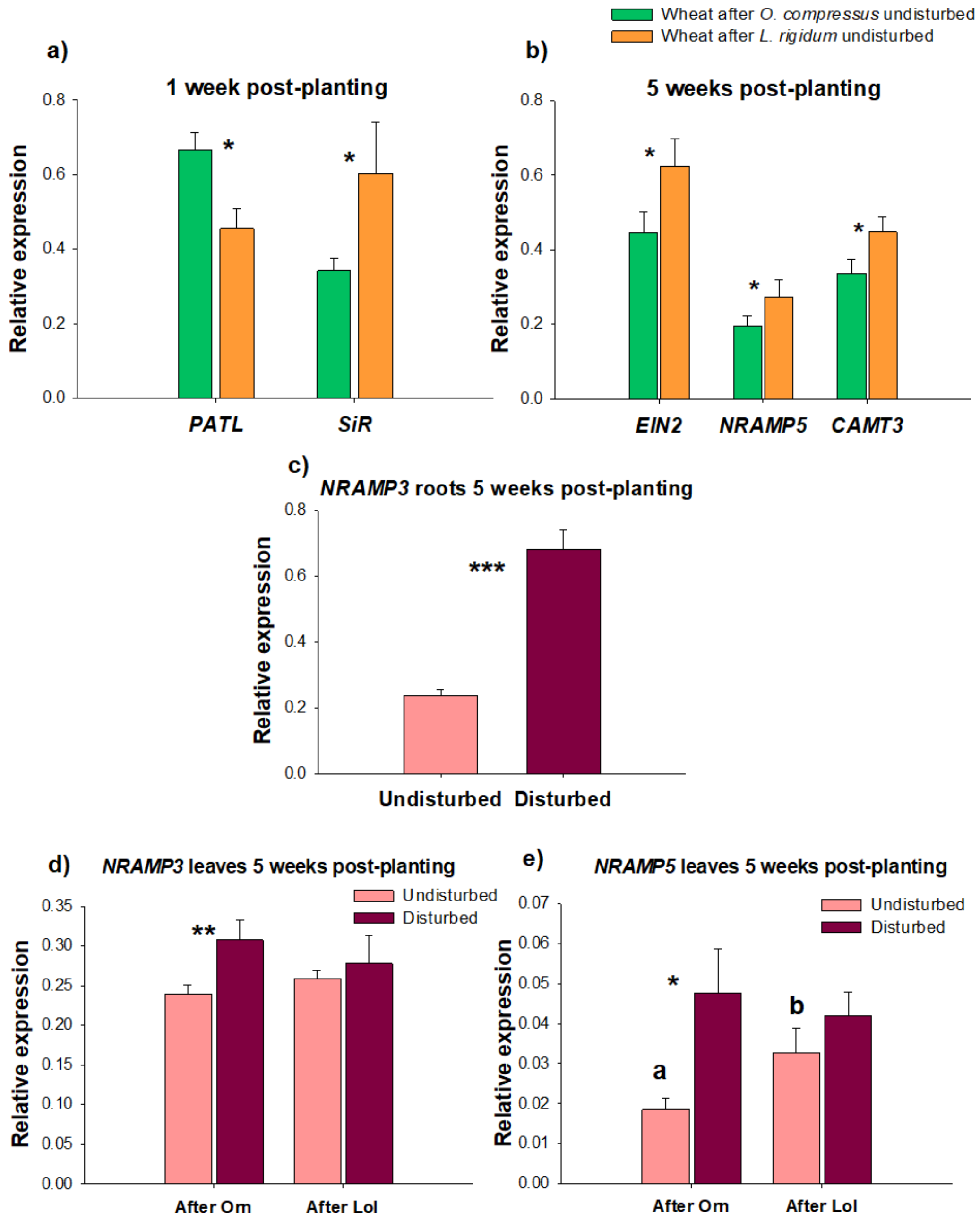

Supplementary Fig. S9. The expression of six wheat genes selected among those differentially expressed between Disturbed and Undisturbed regimes or between *O. compressus* and *L. rigidum* treatments were analysed by qRT-PCR.

Table S1. Primers used for validation of wheat (*Triticum aestivum*) selected genes by RT-qPCR

Gene	Accession #	Forward sequence (5'→3')	Reverse sequence (5'→3')	Amplicon size (bp)	E (%)
PATL	TraesCS1A02G268500.2	ATCGCCAAGAAGGTGTTCATC	AAGAGGGTCTCGGCGGTC	134	102
SiR	TraesCS1A02G323400.1	CGTCTCCACGCCTCTGAAG	CAGCAGTCTCGTTGACATTGG	140	104
CAMT3	TraesCS4D02G304500.3	AAGTCAAGGCTGGAAAATCAAG	ACCGTCTGTACTTTGTGAAGGC	110	100
NRAMP3	TraesCS7D02G451900.1	ACAATATGGGGTCCGTAAACTG	GCCTCGCACAACTTCTGAAG	118	90
NRAMP5	TraesCS4B02G300600.1	TTGGCGTGGTTACAGGAAAG	ACCTCCGCAAGAAGCCAG	91	97
EIN2	TraesCS4D02G036000.3	CCATGATTTCAGGCATAGCAGTTG	CCGAGGGGATAAGAGATATGG	119	104
Act	TraesCS5D02G132200.1	AGTGGACGCACAACAGGTATC	CATCAAACAGTCAGTTAGGTCGC	138	89
Peptidyl-prolyl cis-trans isomerase	TraesCS6B02G093100.2	CACTACAAGGGCAGCTCGTT	AACTTCTCGCCGTAGATGGA	110	97
GAPDH	TraesCS6D02G196300.2	ATTAAGGGTGGTGCCAAGAAG	CAGACTTGTATTCCTTCTCATTGAC	94	95

For each gene, accession number, amplicon size (bp) and amplification efficiency are indicated. The annealing temperature of all primer pairs is 60 °C.

Table S2. Concentration values of Mg, Mn, P, K and Ca on wheat shoots at 5 weeks post-planting.

Treatment	Samples	Mg	Mn	P	K	Ca	Mg/Mn
Undisturbed *O. compressus*	1	2491.877	74.961	2136.747	36932.333	3781.351	33.24
	2	2777.252	86.509	1782.305	34181.187	4497.694	32.10
	3	2889.840	100.853	2154.022	38441.969	4619.767	28.65
Disturbed *O. compressus*	4	2171.168	109.538	735.281	24282.427	2911.190	19.82
	5	2742.591	141.893	838.876	29066.666	3326.379	19.33
	6	2331.476	132.128	800.637	29589.606	3129.916	17.65
Undisturbed *L. rigidum*	7	3065.997	68.386	1864.398	30186.110	5121.524	44.83
	8	3228.353	82.648	1847.791	31990.785	5128.294	39.06
	9	2902.809	77.755	1777.235	39545.710	3945.554	37.33
Disturbed *L. rigidum*	10	2431.807	112.756	696.658	26023.788	3933.681	21.57
	11	2401.700	140.128	815.367	28529.659	3144.122	17.14
	12	1565.599	90.943	693.801	23245.586	1729.663	17.22

Concentrations of cations are given by mg / Kg shoot DW

Table S3. Read numbers obtained from the *Triticum aestivum* transcriptome during the bioinformatic analysis.

Treatment			Library	# raw reads	# reads after trimming	# mapped reads	% of mapped reads
**1 week**	***O. compressus***	Und	1WOU1	29 869 174	29 359 660	24 643 745	83.88
			1WOU2	54 601 724	52 939 464	45 587 590	86.13
		Dis	1WOD1	29 202 166	25 342 166	21 332 897	84.20
			1WOD2	44 430 030	42 683 398	36 500 400	85.49
	L. rigidum	Und	1WLU1	56 065 052	53 666 040	41 005 808	76.42
			1WLU2	48 114 410	46 471 632	37 666 586	81.05
		Dis	1WLD1	36 136 020	35 000 464	30 117 769	86.04
			1WLD2	44 304 746	42 896 220	36 740 543	85.63
**5 weeks**	***O. compressus***	Und	5WOU1	48 660 686	47 183 672	27 561 183	58.45
			5WOU2	37 458 178	35 575 338	21 215 105	59.65
		Dis	5WOD1	13 584 462	10 507 628	6 662 774	63.43
			5WOD2	52 987 650	51 227 340	32 254 489	62.97
	L. rigidum	Und	5WLU1	48 791 424	47 393 518	29 009 663	61.22
			5WLU2	55 838 398	54 231 896	38 561 288	71.16
		Dis	5WLD1	37 588 434	36 747 452	20 672 419	56.21
			5WLD2	42 773 886	41 768 400	24 058 698	57.60

Und – Undisturbed; Dis – Disturbed

Table S4. Read numbers obtained from the *Rhizoglomus irregulare* transcriptome during the bioinformatic analysis.

			Library	# mapped reads	# genes
**5 weeks**	*O. compressus*	Und	5WOU1	225 958	7195
			5WOU2	122 052	3960
		Dis	5WOD1	33 442	975
			5WOD2	223 236	7009
	L. rigidum	Und	5WLU1	315 838	9492
			5WLU2	119 866	5624
		Dis	5WLD1	208 311	6793
			5WLD2	211 960	7107

Und – Undisturbed; Dis – Disturbed
